# Tunable MEMS-based metamaterial nanograting coupler for C-band optical communication application

**DOI:** 10.1186/s11671-023-03843-3

**Published:** 2023-04-25

**Authors:** Kunye Li, Yu-Sheng Lin

**Affiliations:** School of Electronics and Information Technology, Sun Yat-Sen University, Guangzhou, 510006 China

**Keywords:** Metamaterial, Nanograting coupler, MEMS, Optical communication

## Abstract

A tunable metamaterial nanograting coupler (MNC) is presented that is composed of a one-dimensional surface nanograting coupler with a bottom reflector and the metamaterial atop. For a single nanograting coupler, by introducing a reflector and optimizing nanograting parameters, the spatial coupling efficiency exceeds 97% around near-infrared wavelength of 1.43 μm. The metamaterial can be tuned by using micro-electro-mechanical system (MEMS) technique. The relative height or lateral offset between metamaterial and coupling nanograting can be controlled, that the light-emitting efficiency can be separated into two different directions. In addition, the coupling efficiency is as high as 91% at the optical C-band communication window. Therefore, the proposed MEMS-based MNC not only has the possibility of coupling optical fibers with high-density integrated optoelectronic chips, but also has potential application prospects in light path switching, variable optical attenuation, and optical switch.

## Introduction

Metamaterial has extraordinary electromagnetic characteristics that originates from the specific geometric structure arranged artificially and its subwavelength configuration [1]. By changing the geometrical parameters and material structures of metamaterial, a strong electromagnetic response covering the range from visible light [2–5], infrared (IR) wave [6–9] to terahertz (THz) frequency [10–13] can be realized. It has been proved that metamaterial can achieve unique optical characteristics, such as negative refractive index [14], strong transmission [15], perfect absorption [16–19], and holographic imaging [20], due to its exotic permittivity and permeability. To date, metamaterial has been widely applied in various fields such as cloaking [21], sensing [22], detection [23], communication [24], and so on. Without exception, the design scheme of metamaterial used in optical communication is also developing vigorously.

Although metamaterial has excellent electromagnetic properties, the geometric size, position and shape of metamaterial are hardly to be changed when it is fabricated on rigid substrates [25]. The fixed structure and position limit the spectrum range of electromagnetic response of metamaterial. Therefore, the demand of actively tuned metamaterial is generally necessary in the practical application. There have been reported various methods to control metamaterial, e.g., the applying an external electric field [26], using the phase change materials [27, 28], stimulate the laser pumping [29], and driving the mechanical control [30, 31]. Among of these controllable methods, the electromechanically controllable method possesses a wider tuning range of resonant frequency. Micro-electro-mechanical system (MEMS) technique is mature and compatible with complementary metal oxide semiconductor (CMOS) technology [32], thus it is very suitable for the tuning of high-density integrated optoelectronic devices.

The photonic integration is considered as the hotspot research direction in the future. There have been realized various optical elements in silicon photonics, including on-chip light source [33], optical waveguide [34], filter [35], electro-optic modulator [36], wavelength division multiplexer (WDM) [37], optical switch [38], and so on. Nonetheless, to achieve high-efficient light coupling between the chips and optical fibers for silicon photonic chips remains a challenge [39]. The grating coupler provides an efficient solution, and high coupling efficiency is obtained by depositing polysilicon cladding [40], introducing metal mirror [41–43] or distributed Bragg mirror (DBR) [44] and designing apodized grating [45], etc. However, the common grating coupler has a single function that cannot modulate actively. It is extremely restrictive for the multifunctional and high-density integrated optical communication and optical interconnection chip, because it cannot reduce the complexity of the devices and the volume of the chip.

In this study, a tunable MEMS-based metamaterial nanograting coupler (MNC) is proposed to realize flexible and multiple functions. The relationships of nanograting parameters and metamaterial on the coupling efficiency are studied. By changing the relative position of metamaterial and nanograting, the coupling efficiency can be tuned actively. It is expected to realize the integrated functions of optical waveguide, variable optical attenuation, optical switch, and optical path switching to expand the applications of integrated silicon photonics in optical communication and optical interconnection systems.

Designs and methods

**Fig. 1:**
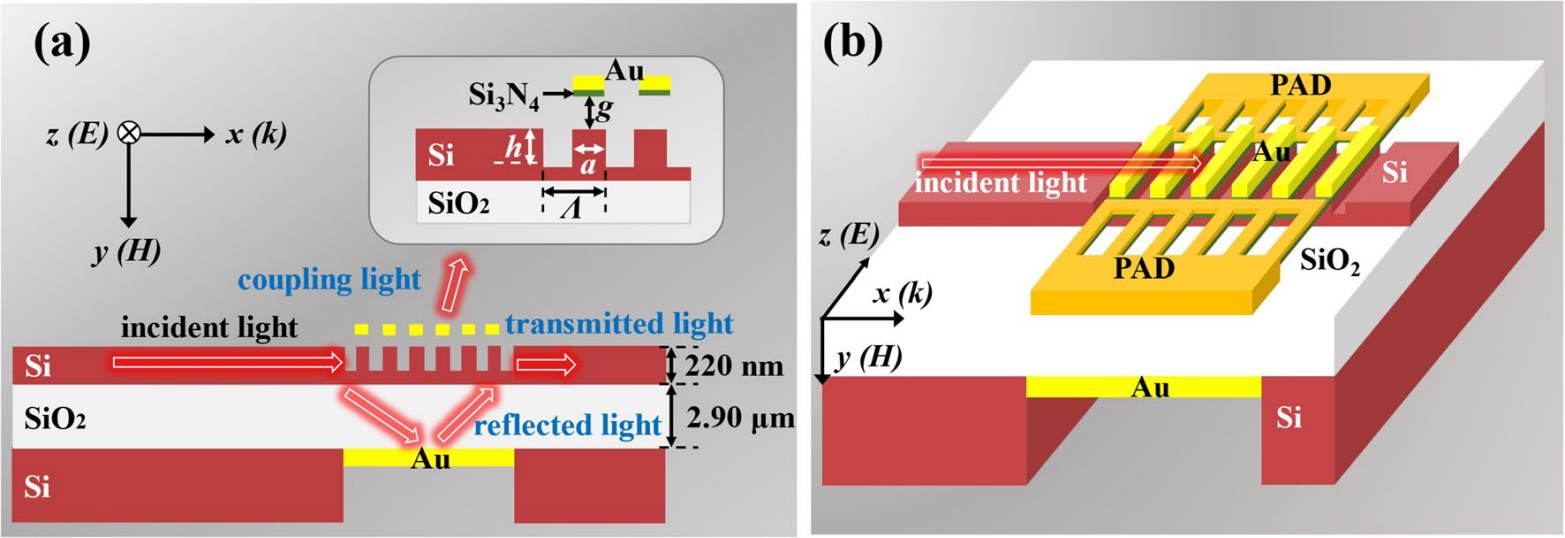
Schematic drawings of MEMS-based MNC for **a** cross-sectional and **b** 3D view, respectively

Figure 1a illustrates the cross-sectional schematic diagram of tunable MEMS-based MNC. The proposed device is composed of a one-dimensional (1D) nanograting coupler with a bottom gold (Au) reflector on Si-on-insulator (SOI) substrate and the metamaterial atop. The nanograting coupler is on the Si device layer with a thickness of 220 nm, and the thickness of the buried oxide (BOX) layer is 2.90 μm. There are three nanograting parameters to be investigated, which are nanograting period (*Λ*), nanograting height (*h*) and nanograting duty cycle (the ratio of nanograting width (*a*) to nanograting period, *a*/*Λ*), respectively. To enhance the directionality, an Au layer is introduced underneath the nanograting to act as a reflector. The incident light propagates along the top Si waveguide along *x*-axis direction, and its electric field vector is parallel to the nanograting (*z*-axis direction). It will be divided into three parts after passing through the nanograting structure as shown in Fig. 1a. First, it is the diffraction light out of free space by nanograting called the coupling light. The transmission intensity ratio of coupling light to incident light is defined as coupling efficiency. Second, the light diffracted into the BOX layer is named reflected light, which will be reflected by the bottom mirror to enhance the directivity of the incident light into Si waveguide. Third, the incident light coupled into Si waveguide will continue to propagate along the *x*-axis direction, which is called transmitted light.

In order to actively modulate the coupling efficiency, an Au metamaterial layer is introduced with a fixed thickness of 10 nm suspended on nanograting structure with a height of *g* value. Figure 1b shows the schematic drawing of three-dimensional (3D) view of MEMS-based MNC (the nanograting length is simplified), metamaterial is supported by the cantilevers composed of a bilayer structure of Si and Si_3_N_4_ to form electrothermal actuators (ETAs) with electrode pads. By driving a DC bias voltage on ETAs, the attraction force can bend the cantilevers upward and downward to the Au nanograting structures owing to the bilayer structure of Si and Si_3_N_4_ have different thermal expansion coefficients. It is considering the Bragg condition (phase matching condition), which gives the relationship between the wavevector (*k*_0_) of incident light and the coupling light in waveguide with m’-order propagation constant ($\beta_{m^{\prime}}$) expressed by [39]

1 $${k}_{0}sin\theta +mG={\beta }_{{m}^{{\prime}}}$$

where *θ* is the diffraction angle, *G* is the grating vector, *m* is the grating diffraction order. Whereas, the Bragg condition does not illustrate the coupling efficiency, nor does it reveal the relationship between grating structure and coupling efficiency. Therefore, in this study, the optical characteristics of MEMS-based MNC are calculated and simulated by using finite-difference time-domain (FDTD) method.

## Results and discussions

**Fig. 2:**
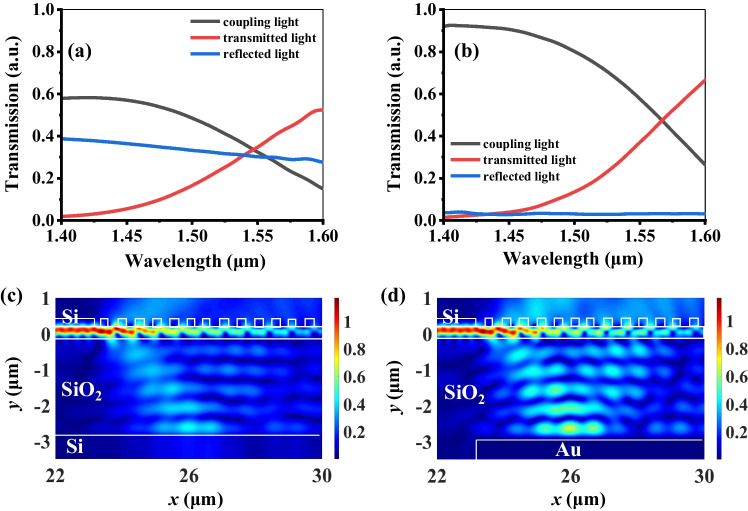
Transmission spectra of nanograting coupler **a** without and **b** with an Au reflector. The electric field distributions monitored at the wavelength of 1.42 μm **c** without and **d** with an Au reflector, respectively

The coupling efficiency of nanograting coupler without metamaterial is optimized first to achieve a large tuning range of transmission intensity. The adapted approach is used an inserted reflector under the nanograting coupler to recycle the propagation power radiated into SiO_2_ layer and then reflected back into Si waveguide to improve the directivity as shown in Fig. 1a. Figure 2a shows the transmission spectra of nanograting coupler without Au reflector along three directions. The maximum coupling efficiency is less than 60%, which is coupling light as black curve shown in Fig. 2a. It is because of some of the incident light propagated to substrate (blue curve) as a reflected light and the rest of the incident light propagated forward (red curve) as a transmitted light. To analyze the energy field distribution of incident light intuitively, the electric field distribution along three directions is illustrated in Fig. 2c. The monitored wavelength is at 1.42 μm. The low coupling efficiency is because the generation of reflected light. Thus, we design an Au reflector underneath the nanograting coupler. It makes the reflected light back into Si waveguide and then enhanced the coupling light. Figure 2b shows the transmission spectra of nanograting coupler with Au reflector along three directions. The maximum value of coupling efficiency is significantly enhanced from 60 to 92% at the wavelength of 1.42 μm, while the reflected light efficiency is evidently decreased from 39 to 2% in the whole C-band wavelength. Meanwhile, the transmitted light efficiency is also improved. These results are attributed to the reflection of the bottom metal reflector. The electric field distribution monitored at the wavelength of 1.42 μm indicates the coupling efficiency is enhanced as shown in Fig. 2d. It proves that the implantation of an Au reflector on the bottom of the nanograting coupler can enormously enhance the coupling efficiency.

**Fig. 3:**
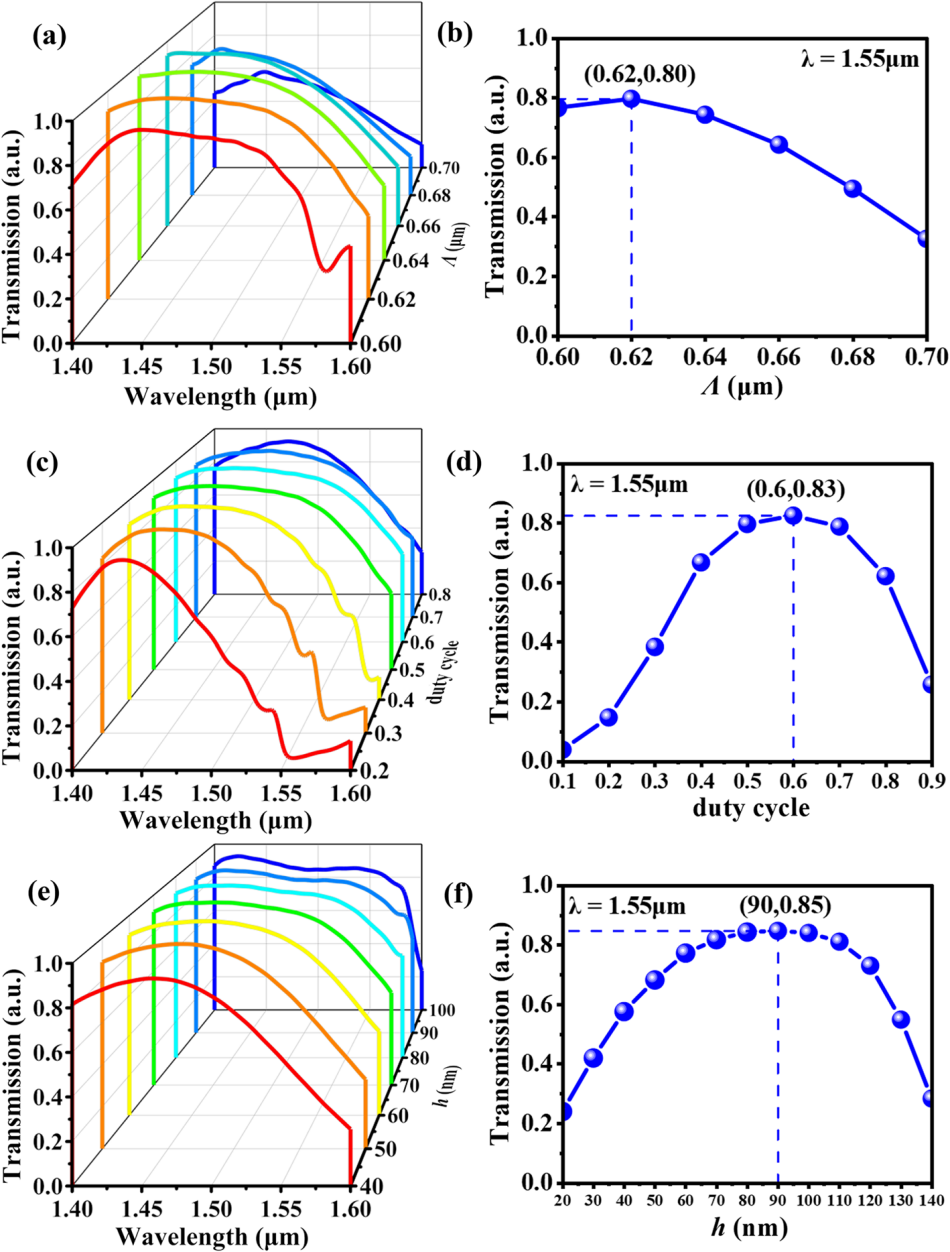
**a**, **c**, and **e** are the transmission spectra of coupling light of nanograting coupler with different nanograting period (*Λ*), duty cycle (*a*/*Λ*), and height (*h*), respectively. **b**, **d**, and **f** are the relationships of **a**, **c**, and **e** monitored at the wavelength of 1.55 μm, respectively. (*λ* represents the wavelength of incident light.)

When the Au reflector is implanted into nanograting, its parameters need to be optimized to achieve the maximum coupling efficiency. Figure 3a shows the transmission spectra of coupling light with different nanograting period (*Λ*) changing from 0.6 to 0.7 μm, while the duty cycle and nanograting height are kept as constant as 0.5 and 70 nm, respectively. When *Λ* = 0.62 μm, the whole spectra band has the better and uniform coupling efficiency from the wavelength of 1.40–1.60 μm, and the average coupling efficiency is over 85%. For the C-band optical communication window, Fig. 3b shows the relationship of transmission spectra and nanograting periods at the incident wavelength of 1.55 μm. The maximum coupling efficiency reaches 80% under the condition of *Λ* = 0.62 μm. Consequently, the nanograting period is fixed as 0.62 μm. For the duty cycle, it can be determined by modifying the nanograting width (*a*). Figure 3c shows the transmission spectra of coupling light with different duty cycle changing from 0.2 to 0.8, while the nanograting period and height are kept as constant as 0.62 μm and 70 nm, respectively. The coupling efficiencies are higher than 90% in the duty cycle range of 0.3 to 0.7 at the wavelength of 1.45 μm. Figure 3d shows the relationship of transmission spectra and duty cycle at the incident wavelength of 1.55 μm. When the duty cycle is 0.6, the maximum coupling efficiency reaches 83%. In comprehensive consideration, the nanograting width (*a*) equals 0.372 μm (0.62 μm × 0.6). For the nanograting height *h*, Fig. 3e shows the transmission spectra of coupling light with different *h* values changing from 40 to 100 nm, while the nanograting period and duty cycle are kept as constant as 0.62 μm and 0.6, respectively. When *h* = 60 nm, the maximum coupling efficiency exceeds 97% around the wavelength of 1.43 μm, and the average coupling efficiency is over 95% at the wavelength range from 1.40 to 1.50 μm, which exhibits the high coupling efficiency spanned the broadband wavelength. As a result, the *h* value is determined as 60 nm. Similarly, the maximum coupling efficiency is 85% at* h* = 90 nm for the incident wavelength of 1.55 μm as shown in Fig. 3f. Eventually, the preferable nanograting parameters are determined as *Λ* = 0.62 μm, *a*/*Λ* = 0.6, *h* = 60 nm, and then metamaterial is introduced to realize tunable MEMS-based MNC.

**Fig. 4:**
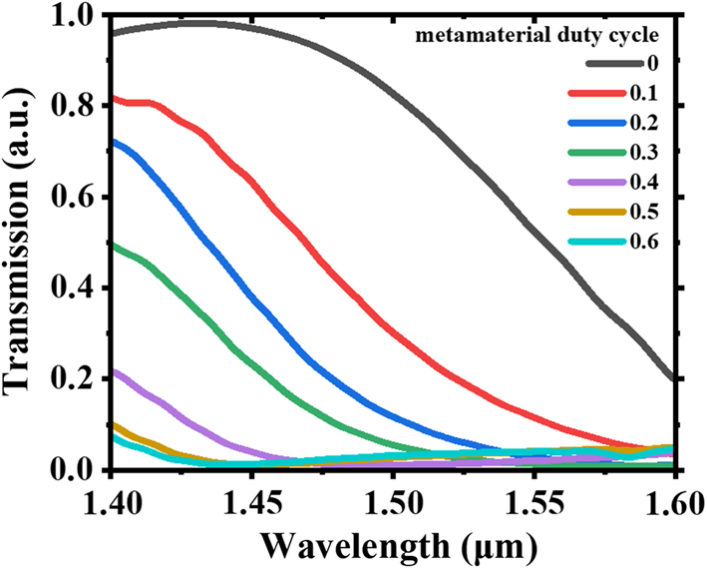
Transmission spectra of MEMS-based MNC with different duty cycle of the metamaterial when the gap between metamaterial and nanograting coupler (*g*) is 0 nm

The metamaterial is implanted on the nanograting coupler as shown in Fig. 1. To obtain wider tuning range of transmission intensity of coupling light, the metamaterial geometric needs to be determined. For convenience, the metamaterial width is expressed as a duty cycle relative to the nanograting period. Figure 4 shows the transmission spectra of MEMS-based MNC with different duty cycle of the metamaterial when the gap between metamaterial and nanograting coupler (*g*) is 0 nm. The coupling efficiency is close to 100% at the wavelength of 1.43 μm when there is no metamaterial as the black curve shown in Fig. 4. It is consistent with the previous results. After implanting metamaterial into nanograting coupler, the coupling efficiency decreases obviously. The duty cycle of metamaterial increases and then the coupling efficiency decreases. When the duty cycle of metamaterial equals to that of nanograting width (*a*/*Λ* = 0.6), the coupling efficiency of the whole band is the lowest. It is 0% around the wavelength of 1.45 μm. These results show that the coupling efficiency of nanograting coupler can be tuned between 100 and 0% without and with the implantation of metamaterial.

**Fig. 5:**
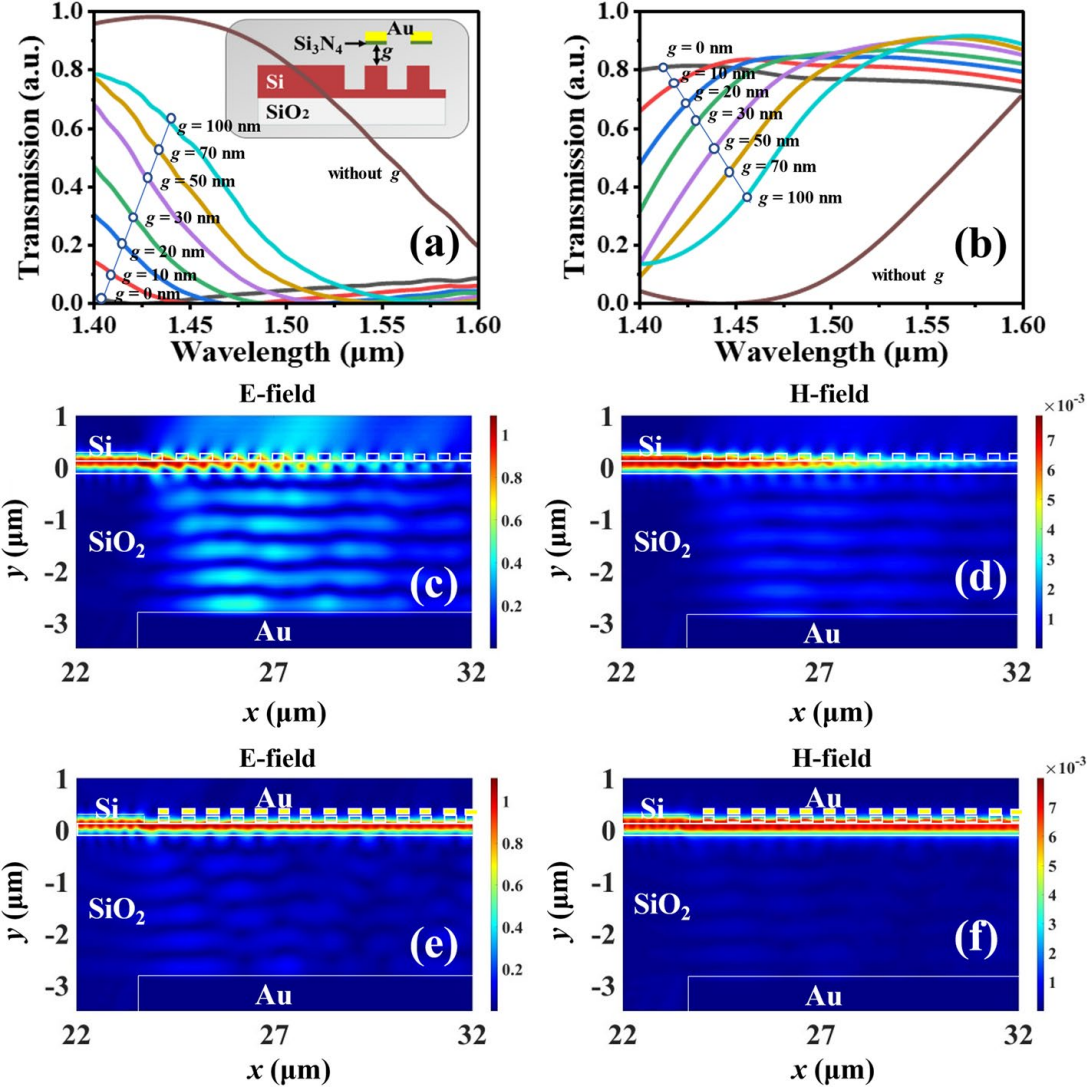
Transmission spectra of **a** coupling light and **b** transmitted light of MEMS-based MNC with different metamaterial height (*g*) values. **c** Electric and **d** magnetic fields distributions of MEMS-based MNC without metamaterial at the wavelength of 1.45 µm. **e** Electric and **f** magnetic fields distributions of MEMS-based MNC with metamaterial when *g* = 0 nm at the wavelength of 1.45 µm

As above-mentioned, metamaterial can affect the coupling efficiency of coupling grating, which can be tuned by changing *g* value. Figure 5a shows the transmission spectra of coupling light of MEMS-based MNC with different *g* values. When *g* = 0 nm, the coupling efficiency of whole band is very low, and it is 0% around the wavelength of 1.45 μm. Along with the increment of *g* value from 0 to 100 nm, MEMS-based MNC exhibits the characteristics of tunable coupling efficiency and wavelength dependence in the C-band spectrum range. According to this trend, it can be identified that when *g* value increases to infinity, it is equivalent to the absence of metamaterial, and then the coupling efficiency is the same as that without metamaterial as the brown curve shown in Fig. 5a. Moreover, Fig. 5b shows the transmission spectra of transmitted light under the same conditions of Fig. 5a. It exhibits the opposite trend with coupling efficiency in the wavelength range from 1.40 to 1.50 μm. When *g* = 0 nm, the transmitted light is maximum up to 80%. The reasons for that being not 100% are attributed to the propagation loss and material absorption. Therefore, the coupling efficiency and transmitted light intensity can be controlled by changing *g* value. The design provides an effective approach for the use in variable light attenuation, optical switching, and optical path switching applications. In order to intuitively comprehend the physical mechanism of electromagnetic responses of nanograting coupler and metamaterial to incident light, Fig. 5c–f show the electric and magnetic field distributions at the wavelength of 1.45 μm when the MEMS-based MNC without (Fig. 5c, d) and with (Fig. 5e, f) metamaterial atop and *g* = 0 nm, respectively. In Fig. 5c, d, the incident light is diffracted by the nanograting coupler and then reflected by the bottom mirror. Most of incident light will emit into free space, and then there has no light energy can be continuously propagated forward. Through the field distributions, the electric (E) field intensity is higher than the magnetic (H) field intensity, which indicates that the electric field plays a major role than magnetic field. As a result, the coupling efficiency is 100% and the transmitted light efficiency is 0%. In Fig. 5e, f, the metamaterial suppresses the diffraction of nanograting coupler into free space, which makes the incident light continuously propagates through Si waveguide. The coupling efficiency is 0% and the transmitted light efficiency is 80%.

**Fig. 6:**
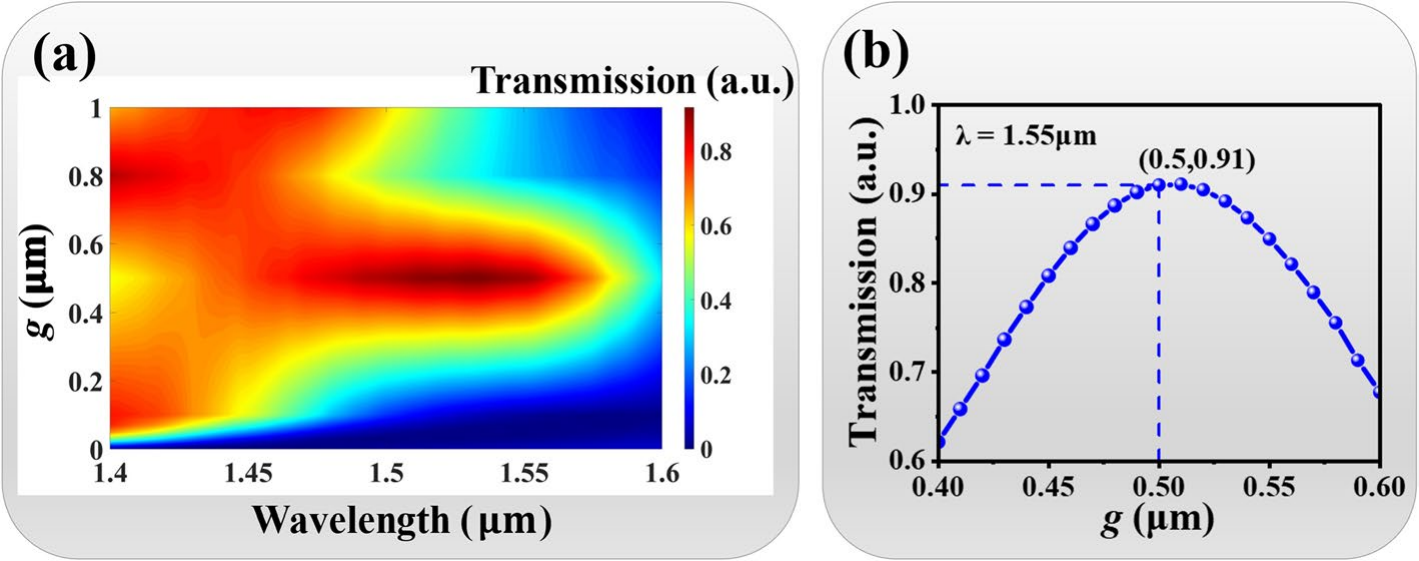
**a** Transmission intensity distribution of coupling light of MEMS-based MNC by changing *g* value from 0.1 to 1.0 μm. **b** Relationship of transmission intensity of coupling light and *g* values at the wavelength of 1.55 μm

Figure 6a shows the transmission intensity distribution of coupling light of MEMS-based MNC by enlarging *g* over 0.1 μm. When *g* value is larger than 0.1 μm, the wavelength corresponding to the maximum coupling efficiency of the coupling light can be shifted. It is first red-shifted when *g* value smaller than 0.5 μm and then gradually blue-shifted when *g* value larger than 0.5 μm. Under the condition of *g* = 0.5 μm, the maximum coupling efficiency of the coupling light is at the wavelength of 1.55 μm. Figure 6b plots the relationship of the transmission intensity of coupling light and *g* value when the incident wavelength is 1.55 μm. The coupling efficiency increases first and then decreases by increasing *g* value. The maximum transmission intensity is 91%. This means that when the metamaterial elevates 0.5 μm, the coupling efficiency of C-band optical communication window can be appropriately improved.

**Fig. 7:**
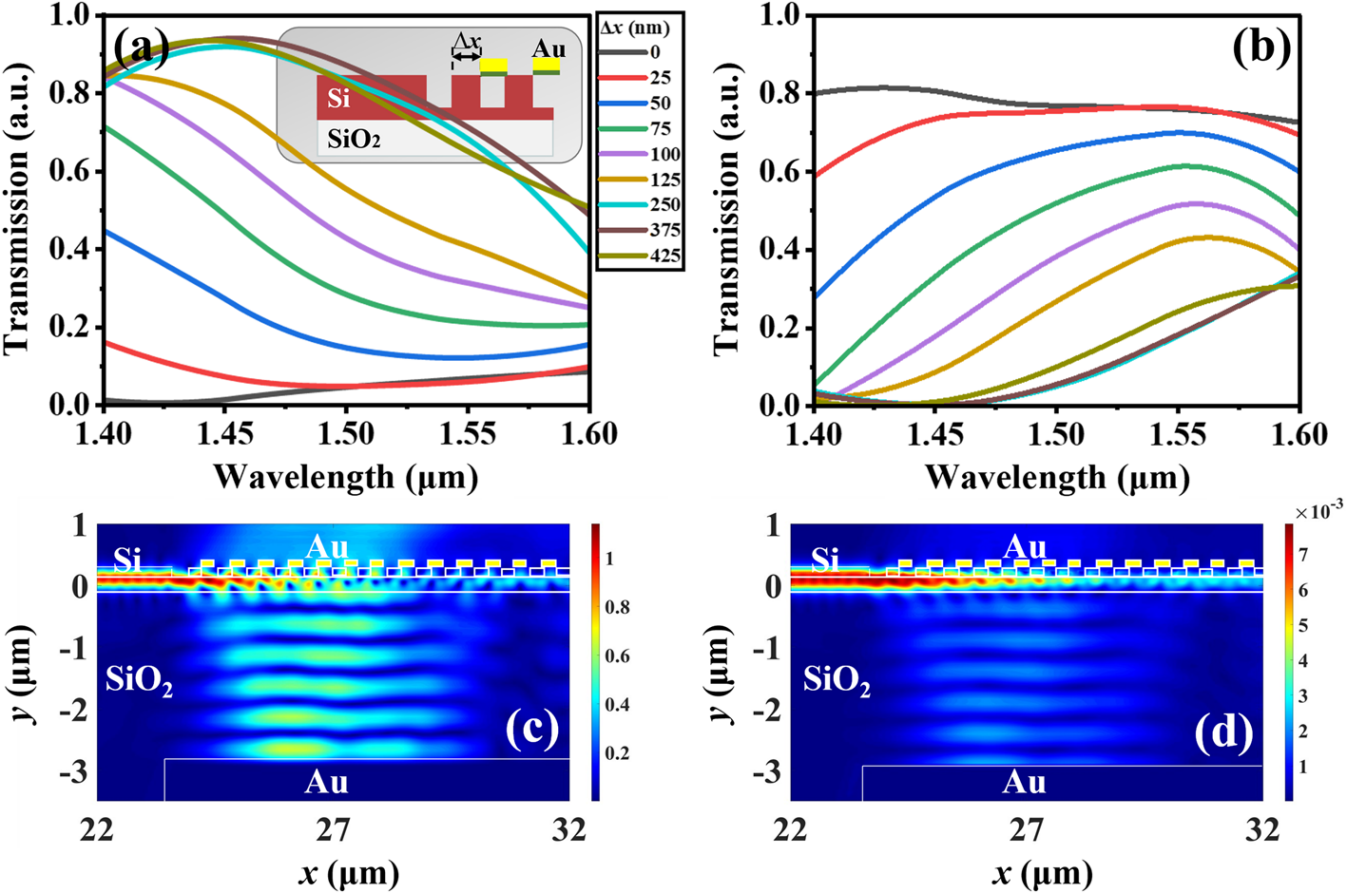
Transmission spectra of **a** coupling light and **b** transmitted light of MEMS-based MNC with different *∆x* values when *g* = 0 nm, respectively. **c** Electric and **d** and magnetic fields distributions when *∆x* = 375 nm at the wavelength of 1.45 µm, respectively

Furthermore, the coupling efficiency is also affected by the lateral offset of metamaterial relative to nanograting coupler. The assumed condition is *g* equals 0 nm to discuss the influence of lateral offset of metamaterial to coupling efficiency. Figure 7a shows the transmission spectra of coupling light of MEMS-based MNC with the lateral offset of metamaterial, which defines as *∆x*. Since the nanograting coupler has periodicity, the lateral offset is less than one period of nanograting coupler. When *∆x* = 0 nm, the coupling efficiency is very low and it is close to 0% around the wavelength of 1.45 μm. It can be enhanced by enlarging *∆x* value. When *∆x* = 375 nm, the coupling efficiency of the whole band is the optimum, and its maximum value is over 90%, however, it deteriorates when *∆x* is larger than 375 nm. Therefore, by changing *∆x* value, the coupling efficiency of nanograting coupler will increase and then decrease. There exists a maximum coupling efficiency. Similarly, the transmitted light efficiency shows a reversed trend to the coupling efficiency as shown in Fig. 7b. When *∆x* equals 0 nm, the transmitted light efficiency is the maximum, which is up to 80% and is in accordance with the previous results. To explain these electromagnetic responses well, Fig. 7c, d illustrate the electric and magnetic fields distributions of MEMS-based MNC with *∆x* = 375 nm at the wavelength of 1.45 µm. It can be observed that the inhibition of metamaterial on nanograting diffraction is weak when *∆x* equals 375 nm, which is equivalent to the nanograting coupler without metamaterial. In consequence, the coupling efficiency and transmission light intensity in two directions can be controlled by changing the lateral offset of metamaterial relative to nanograting coupler.

## Conclusion

In conclusion, a tunable MEMS-based MNC is presented by integrating a 1D surface nanograting coupler and metamaterial atop. Through the implantation of reflector and the optimization of nanograting parameters, the coupling efficiency can be great improved. Therefore, a relatively large tuning range of coupling efficiency can be obtained. Using MEMS technique to change the relative height or lateral offset of metamaterial and nanograting coupler can realize the tunable transmission intensity and light-emitting direction, and also enhance the coupling efficiency at the C-band optical communication window appropriately. The proposed device improves the flexibility of nanograting coupler to have further applications in photonic integration, optical communication, and optical interconnection. It is expected to realize various functions such as optical waveguide, variable optical attenuation, optical switch, and so on.

## Data Availability

All data generated or analyzed during this study are included in this published article.
